# New developments in adult congenital heart disease

**DOI:** 10.1007/s12471-020-01455-5

**Published:** 2020-08-11

**Authors:** B. J. Bouma, G. T. Sieswerda, M. C. Post, T. Ebels, R. van Kimmenade, R. J. de Winter, B. J. Mulder

**Affiliations:** 1 Department of Cardiology, Amsterdam UMC, University of Amsterdam, Heart Center, Amsterdam Cardiovascular Sciences, Amsterdam, The Netherlands; 2 Department of Cardiology, University Medical Center Utrecht, Utrecht, The Netherlands; 3 Department of Cardiology, St Antonius Hospital, Nieuwegein, The Netherlands; 4 Department of Cardiothoracic Surgery, University Medical Center Groningen, University of Groningen, Groningen, The Netherlands; 5 Department of Cardiology, Radboud University Medical Center Nijmegen, Nijmegen, The Netherlands

**Keywords:** Congenital heart disease, GUCH, Adults, New developments, Cardiac surgery, Interventions

## Abstract

Congenital heart disease (CHD) affects 0.8% of live births and over the past decades technical improvements and large-scale repair has led to increased survival into adulthood of over 95% of the new-born. A new group of patients, those who survived their congenital heart defect, has emerged but late complications including heart failure, pulmonary hypertension (PH), arrhythmias, aneurysms and endocarditis appeared numerous, with a huge impact on mortality and morbidity. However, innovations over the past years have changed the landscape of adult CHD dramatically. In the diagnostic process important improvements have been made in the use of MRI, biomarkers, e‑health concepts and 3D visualisation of anatomy. Care is now concentrated in specialised centres, with a continuous emphasis on education and the introduction of weekly multidisciplinary consultations on diagnosis and intervention. Surgery and percutaneous intervention have been refined and new concepts applied, further reducing the burden of the congenital malformations. Research has matured from case series to global networks. Currently, adults with CHD are still facing high risks of early mortality and morbidity. By global collaboration and continuous education and development and innovation of our diagnostic and therapeutic arsenal, we will improve the perspectives of these young patients.

## Dutch contribution to the field

- Excellent care for adult patients with congenital heart disease.
- Establishment of a national registry and DNA bank (CONCOR).
- Important contribution in scientific research in adult congenital heart disease.
- Leading role in global research networks.

## Introduction

**Fig. 1:**
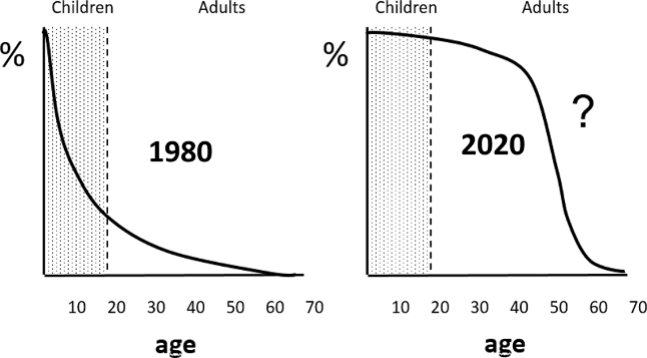
Uncertainty of clinical course of adults with congenital heart disease

Birth prevalence of congenital heart disease (CHD) is estimated to be 8 cases per 1000 live births and up to 95% survive until adulthood (Fig. 1; [1]). A decline is to be expected as nowadays up to one third of the defects and 57% to 85% of all severe lesions are detected before the end of the pregnancy, next to prevention by the prescription of vitamin supplements.

**Fig. 2:**
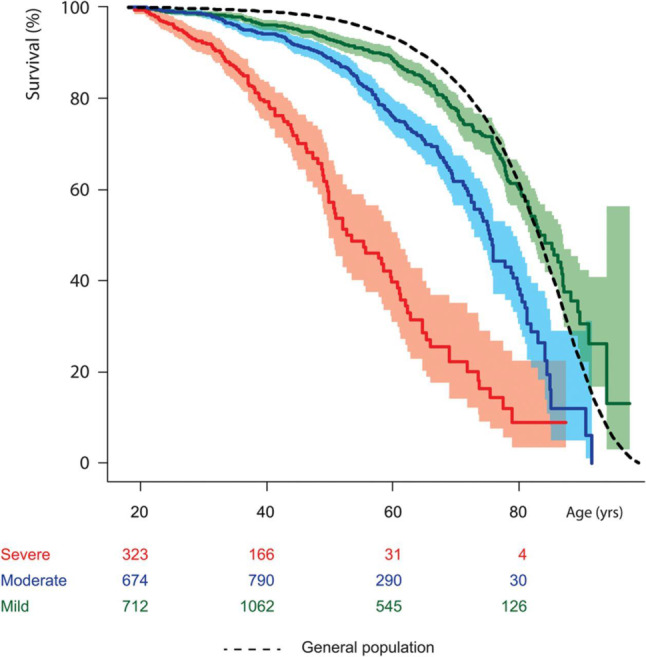
Estimated survival from the age of 18 of adults with congenital heart disease stratified for disease severity

**Fig. 3:**
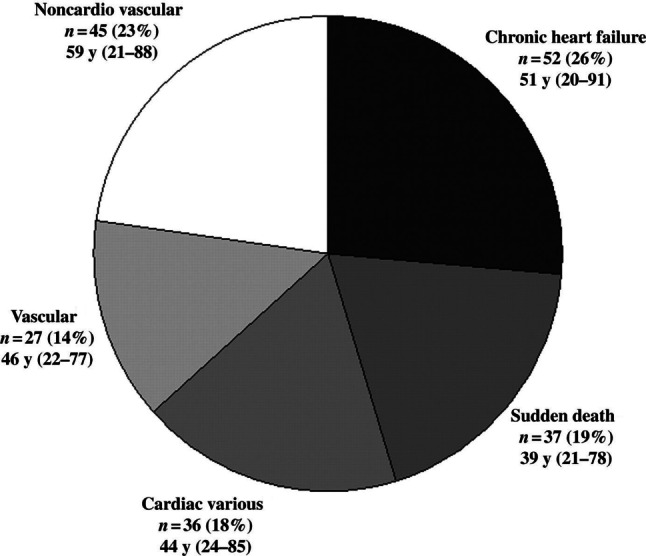
Causes of death of adults with congenital heart disease

Data from the Dutch National CONCOR registry show that survival of adults with lesions of moderate and severe complexity (Fig. 2) is limited compared with the general population and two thirds of the patients die from a cardiac cause (Fig. 3); [2]). Sex differences are common in adult congenital heart disease (ACHD) [3]. Male CHD patients also have a greater risk of endocarditis, aortic events, and indications for internal cardiac defibrillator implantation, while women tend to be more at risk of pulmonary hypertension (PH). The 30-day in-hospital surgical mortality for CHD appeared to be higher in young men than in women [4]. It has been demonstrated that adult men with an atrial septal defect type 2 have a worse survival compared with females [5].

Over the past decades, awareness has grown about the need to deliver appropriate care in patients with end-stage CHD [6]. There are several important end-of-life issues, such as how to cope with a poor prognosis when, early in life, a complete repair and normal lifespan was expected. Crucial components in the end-of-life process should be discussed during the clinical course, e.g. switching off the ICD and appointing a family member as a healthcare representative.

## Diagnostics

Next to echocardiography (3D and strain) and computed tomography (low radiation and high resolution), MRI has emerged over the past few decades as the superior imaging modality for complex ACHD patients. Rapid, high-resolution imaging of complex anatomy and accurate quantitative assessment of physiology and function have become possible [7]. New techniques, including flow quantification, 3D angiography, perfusion imaging and assessment of myocardial viability have become available. Nowadays, our understanding of pathophysiology has increased, for example insight into flow patterns, wall shear stress and energy loss in several clinical conditions such as bicuspid aortic valve, post-coarctectomy haemodynamics and the Fontan circulation.

Upcoming techniques are 3D printing, in which computed tomography or MRI data may be used to reconstruct the heart and vessels [8]. Another promising technique is computer-generated real-time digital holography. Because current 3D images are displaced as a single plane on a 2D screen, it precludes direct interaction and hampers the perception of depth and spatial relationships [9]. These models can be used to precisely visualise complex anatomy, plan surgical procedures, and teach trainees and patients.

Biomarkers are becoming increasingly used in ACHD as clinicians start to recognise the diagnostic and prognostic significance. Brain natriuretic peptide, a cardiac hormone secreted by cardiac myocytes, responds to ventricular wall stress secondary to volume and pressure overload and is elevated in most adults with complex CHD [10]. Troponin, biomarkers of myocardial damage and soluble suppression of tumorgenicity 2, a biomarker of myocardial fibrosis, have prognostic value in ACHD patients with chronic heart failure (HF) and PH. Although the precise position of biomarkers still needs to be determined, they are increasingly being incorporated in daily clinical practice for risk stratification and guidance of therapy.

E‑health is an upcoming modality with high potential. E‑health is a concept covering several processes from telemedicine, teleradiology, teleconsulting, monitoring vital signs, to educational websites. The greatest impact seems to be in the early diagnosis of complications. In ACHD patients it has been shown that e‑health is feasible with high adherence enabling early detection of arrhythmias, hypertension and HF. This may lead to a swift therapeutic response or remote reassurance in case of normal findings [11].

## Late complications

The outstanding improvements in surgical and interventional techniques over the last decades have resulted in a substantial decrease in mortality and morbidity of the ACHD population. This result has evolved from a strategy of not only restoring anatomy, function or haemodynamics, but also from preventing and solving long-term complications. Most complications are related to valvular dysfunction and fibrosis as a result of longstanding volume or pressure overload and the intervention itself, while endocarditis remains relevant. Complications of the modern Western lifestyle such as atherosclerosis, diabetes and obesity need to be taken into account as well.

HF phenotypes in ACHD can be categorised into those with uncorrected defects, a repaired or palliated status and a failing single ventricle physiology [12]. While in acquired HF a broad arsenal of evidence-based medical therapy is available, clinical evidence on left and especially right ventricular failure in ACHD is sparse. In end-stage HF, there is limited experience with mechanical support and transplantation. Restricting factors may be anatomy, presence of PH or immunological factors due to multiple transfusions during previous surgery [12].

Arrhythmogenesis is induced by a myriad of variables including anatomical defects, surgical scarring, hypoxaemia, haemodynamic sequelae and genetic factors [13]. Any new-onset or worsening of arrhythmia should be of concern and prompts haemodynamic assessment [14]. The interaction between ACHD and electrophysiology has resulted in a steep increase of introduction of mapping techniques, ablation and device therapies in ACHD.

Despite medical advances, the prevalence of endocarditis did not decrease [15]. Although the number of cyanotic patients has reduced, more and more patients receive prosthetic materials, where especially percutaneous pulmonary valve prostheses are associated with increased endocarditis risks [16].

ACHD patients are not exempt from acquired cardiovascular diseases. Compared with the general population, the relative risk for developing coronary artery disease (CAD) is between 4.6 and 12.0 for men and women aged 20 years, respectively [17]. Not only the presence of CAD, but also comorbidity such as diabetes, obesity and renal impairment, now play a role in timing and decision-making in ACHD.

## Pulmonary hypertension

PH is a relatively common complication in CHD as approximately 4–10% will develop PH with an incidence of 35% in the elderly [18]. It significantly affects mortality; for example, in Eisenmenger syndrome mortality is severely impaired and morbidity limits exercise capacity and decreases quality of life [19, 20].

Prevention of pulmonary arterial hypertension (PAH) is of paramount importance. Significant shunts should be closed with surgical or transcatheter repair shortly after diagnosis. Follow-up of closed defects is obligatory to detect PAH in an early phase.

Treatment of PAH in CHD should be a multidisciplinary team approach and individualised for each patient according to the underlying aetiology, haemodynamics and clinical status. Supportive therapy is mainly given to avoid or treat complications related to PH-CHD and includes diuretics to unload the right ventricle, oxygen administration, psychosocial support, and supervised rehabilitation.

In the last decades, several drugs interfering with one of the three pathways leading to pulmonary vascular remodelling have become available. In the past, endothelin receptor antagonists, e.g. bosentan, have shown favourable outcome in exercise capacity and pulmonary haemodynamics in patients with Eisenmenger syndrome [21]. However, in a recent randomised study in Eisenmenger patients, macitentan did not show superiority over placebo on improvement in the six-minute walk distance [22]. Interestingly, in a large placebo controlled study, macitentan significantly reduced the composite endpoint of morbidity and mortality in PAH, including CHD [23]. Secondly, phosphodiesterase type 5 inhibitors, such as sildenafil, and guanylate cyclase stimulators, both result in vasodilatation of the pulmonary vasculature and are approved for the treatment of PAH. Recently, in a post-hoc analysis in the subgroup of CHD-PAH patients, an oral selective prostacyclin receptor agonist selexipag showed a delay in disease progression [24]. Nowadays, upfront combination therapy with at least two different PH-specific drugs has become standard when initiating PAH-specific therapy and treatment should include a goal-oriented approach. In patients who are highly symptomatic (functional class IV) intravenous epoprostenol showed favourable effects on exercise capacity, although central lines increase the risk of paradoxical embolism and sepsis in patients with Eisenmenger syndrome [25].

## Percutaneous interventions

**Fig. 4:**
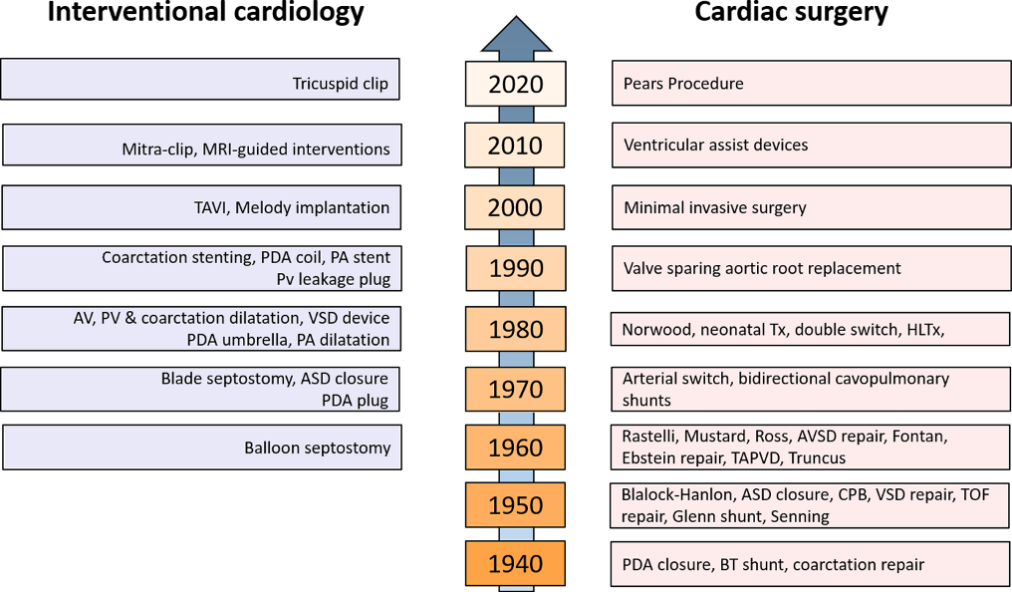
Developments over the years in cardiac surgery and interventional cardiology. *MRI* magnetic resonance imaging, *TAVI* transcatheter aortic valve implantation, *PDA* patent ductus arteriosus, *PA* pulmonary artery, *AV* aortic valve, *PV* pulmonary valve, *VSD* ventricular septal defect, *ASD* atrial septal defect, *Pears* Personalized External Aortic Root Support, *Tx* cardiac transplantation, *HLTx *heart-lung transplantation, *AVSD* atrioventricular septal defect, *TAPVD* Total anomalous pulmonary venous drainage, *CPB* cardiac pulmonary bypass, *TOF* tetralogy of Fallot, *BT* Blalock-Taussig

In the last decade, development of percutaneous techniques has significantly contributed to the management of patients with ACHD (Fig. 4; [26]), Traditionally, simple percutaneous device closure of atrial septal defects type 2 in children and adults have been the treatment of choice over surgery (when feasible regarding anatomy; size, aortic rim). It is expected that the number of patients treated for closure of patent foramen ovale (PFO) for cryptogenic stroke will increase after recent meta-analyses of randomised trials showed significant clinical benefit [27]. Percutaneous balloon valvuloplasty for congenital pulmonary and aortic stenosis have shown reliable and long-lasting results. Device closure of patent ductus arteriosus in children and adults has long been common practice and is recommended as first-choice method of closure if technically feasible.

Percutaneous treatment of native coarctation and re-coarctation in adolescents and adults with stents is the first choice in many centres. Upper limb hypertension and a pressure gradient >20 mm Hg or a stenosis >50% on imaging are the criteria for intervention. Stent placement for pulmonary artery stenosis, as stand-alone procedure or as hybrid procedure with direct visual stent placement during surgical pulmonary valve replacement, is feasible and shows good results [28]. Transcatheter valve implantation for pulmonary valve regurgitation or stenosis in patients with pulmonary conduits (after surgical treatment for Tetralogy of Fallot or Ross procedures) has significantly changed the outlook for these patients. Life-time strategies with alternating surgical and percutaneous valve implantation (and valve-in-valve) have emerged, with both the Melody valve (Medtronic) and Edwards valve (Edwards lifesciences) showing excellent and lasting results [29]. Valve endocarditis after percutaneous implantation remains a concern [30]. Timing of pulmonary valve replacement in asymptomatic patients with signs of right ventricular enlargement, reduced ejection fraction or arrhythmias remains challenging and requires a multidisciplinary approach in dedicated, highly specialised centres.

Future perspectives include the development of bioresorbable devices for ASD/PFO closure [31]. In addition, quality standards and volume requirements will be established for centres that provide care for adults with CHD [32]. These will include formal training for adult interventional cardiologists in ACHD interventions, close collaboration with paediatric cardiologists (joint intervention teams), surgeons, imaging cardiologists, electrophysiologists and multidisciplinary decision-making (Grown-Up Congenital Heart disease/GUCH heart team). Interaction with interventional cardiologists involved in structural heart disease will enhance skills and options for ACHD interventions. Techniques from transcatheter aortic valve implantation (TAVI) programs and the application of mitraclips for mitral and tricuspid regurgitation will be translated to use in ACHD patients, as will new percutaneous valves, annuloplasty devices, plugs for paravalvular leak closure etc.

## Cardiac surgery

Personalised external aortic root support (PEARS) is a low-risk surgical procedure to strengthen the aortic root in connective tissue disorders such as Marfan’s syndrome and probably also degenerative disorders (Fig. 4; [33]). Long-term results are promising, albeit that the number of procedures is limited. Interestingly, this PEARS support also seems to work well in the Ross procedure, where the variable dilatation of the pulmonary autograft under aortic pressures can be prevented by the same support.

Valve sparing root replacement has added considerably to the decrease in long-term morbidity of patients with congenital aortic valve pathology [34]. The longevity of this repair not only depends on the technique, but also on the skills of the surgeon as the pathology is very variable. Because these patients are often young, repeat procedures must be anticipated. This calls for workshops to educate surgeons in this rather artful procedure.

Interestingly, all varieties of prosthetic valves designed for the aortic valve perform worse in the pulmonary position [35]. Homografts for pulmonary valve disease have proven to have the absolute best prognosis, the paucity of their availability being currently the major drawback. As late pulmonary valve replacement by transcatheter valves has shown to be associated with continuing risk of endocarditis of 1–3% per year, surgical solutions associated with a minimum of long-term morbidity are of continued interest [30]. We should strive for a pulmonary valve prosthesis specifically designed for that very position [36].

Late Fontan failure including the elevated central venous pressure is mostly caused by increasing pulmonary resistance due to the un-physiological non-pulsatile flow. Nonetheless, the solution has proven to be cardiac transplantation, after which pulmonary re-remodelling may well occur. A few cases have been described with promising results with cardiac transplantation, also unusual vascular anatomical situations call for lowering the threshold for transplantation [37]. These referrals should also be prompted by hepatic disorders preceding prohibitive cirrhosis or malignancy due to abdominal congestion caused by the chronic low output and high central venous pressure state.

## Clinical research

Research on ACHD has changed dramatically during the past decades from case series and retrospective studies to international prospective cohorts and randomised controlled clinical trials, and from chromosomal studies to whole-genome sequencing. Our national registry (CONCOR) and international networks such as the ISACHD and the EuroGUCH facilitate worldwide collaborative ACHD research and education. Several international initiatives have been undertaken to improve efficacy, such as the NOTE Registry, a global study assessing the efficacy and safety of non-vitamin K oral antagonists in preventing thromboembolic complications in ACHD and the ROPAC study, describing the outcome of pregnancy in cardiac patients, including ACHD [38, 39]. Much of this research is integrated in the 2020 European Cardiac Society guideline on adult CHD. Moreover, the several clinical recommendations on staff and institutional requirements to run an ACHD program have been published [40]. Due to these initiatives, specialised medical care was increasingly delivered to adult patients with CHD, resulting in a dramatic increase of referral to the specialised ACHD centres and a significant reduction in mortality and morbidity in patients with ACHD.

## Conclusion

In the past decades tremendous progress has been made in ACHD care. However, we are facing new challenges to further reduce early mortality and morbidity. By applying improvements in diagnostics and innovations in our therapeutic arsenal together with global collaboration and education, our ACHD patients may benefit from these future achievements.
